# Tetracycline Inhibits Local Inflammation Induced by Cerebral Ischemia via Modulating Autophagy

**DOI:** 10.1371/journal.pone.0048672

**Published:** 2012-11-07

**Authors:** Yongjun Jiang, Juehua Zhu, Li Wu, Gelin Xu, Jianwu Dai, Xinfeng Liu

**Affiliations:** 1 Department of Neurology, Jinling Hospital, Nanjing University School of Medicine, Jiangsu Province, China; 2 Key laboratory of Molecular Developmental Biology, Institute of Genetics and Developmental Biology, Chinese Academy of Sciences, Beijing, PR China; University of North Dakota, United States of America

## Abstract

**Background:**

Tetracycline exerts neuroprotection via suppressing the local inflammation induced by cerebral ischemia. However, the underlying mechanism is not completely clear.

**Methodology/Principal Findings:**

The mRNA and protein expressions of tumor necrosis factor α and interleukin 6 and the number of activated microglia were measured to detect the inflammatory process in the ischemic hemisphere. The key proteins of nuclear factor kappa B pathway and the binding activity of nuclear factor kappa B were also measured. Two key components of autophagy, Beclin 1 and LC3, were detected by western blotting. Pretreatment with tetracycline inhibited the mRNA and protein expressions of tumor necrosis factor α and interleukin 6 and decreased the numbers of activated and phagocytotic microglia. Tetracycline down regulated the total and phosphorylated expressions of IKK, IκB and p65 (*P*<0.05). The autophagy inhibitor, 3-methyladenine, inhibited inflammation and activation of nuclear factor kappa B pathway. The levels of Beclin 1 and LC3 were decreased by 3-methyladenine and tetracycline.

**Conclusions/Significance:**

Our data suggested that pretreatment of tetracycline may inhibit autophagy in the ischemic stroke brain and then suppress the inflammatory process via inhibiting the activation of nuclear factor kappa B pathway.

## Introduction

Inflammation has an essential role in the pathophysiology of ischemic stroke [1]. Ischemia activates the nuclear factor kappa B (NF-κB) pathway and promotes its DNA binding activity, which in turns leads to many inflammatory processes. The activated NF-κB binds to the specific sites in the promoters of its target genes and regulates the expressions of these genes such as tumor necrosis factor α (TNF-α) and interleukin 6 (IL6) [2] which deteriorate the ischemic injury as pro-inflammatory cytokines [3]. Microglia, which is the local inflammatory cell in the brain, could be also motivated by NF-κB and exacerbate the ischemic injury via secreting pro-inflammatory cytokines (such as IL 6 and TNF-α), chemokines and adhesion molecules [4], [5]. On one hand, NF-κB modulates several inflammatory processes, on the other hand, the dynamic and function of NF-κB are under the control of other process. Beclin 1, a critical component in formation of autophagy, is related with NF-κB pathway which indicates autophagy may play a role in the inflammation [6].

Autophagy is a catabolic and important cellular process for organism health. To remove damaged cellular organelles or regenerate metabolites in response to stress, cytoplasmic components and organelles are engulfed by phagophore and emerged as autophagosome which fuses with lysosomes to form autophagolysosomes for bulk degradation [7]. Though suitable autophagy may provide neuroprotection [8], the excessive or inappropriate activation of autophagy could induce cell death [9]. Therefore, inhibiting autophagy is a potential target of stroke treatment [10]. Autophagic activities are significantly increased by focal cerebral ischemia mainly in the border of the lesion where inflammation occurs [11], [12]. Previous research demonstrated that autophagy modulates inflammation via activating NF-κB pathway [13].

Tetracycline is a wide-used and well-tolerated antibiotic in clinics. Recently, minocycline, a semisynthetic second-generation tetracycline, was found to extend the therapeutic time window in experimental stroke [14] and improve the neural function of ischemic stroke patients [15]. The possible mechanism is that tetracycline has the ability to suppress the inflammation after stroke via down-regulating the level of pro-inflammatory cytokines and inhibiting the activation of microglia [16]. Whether tetracycline inhibits local inflammation through suppressing activation of NF-κB pathway via modulating autophagy is unknown.

The present study was aimed to investigate whether pretreatment of tetracycline could inhibit autophagy in the ischemic stroke brain and then suppress the inflammatory process via inhibiting the activation of NF-κB pathway.

## Results

### 2.1 Tetracycline Decreased the Protein Levels of LC3 and Beclin 1

**Figure 1:**
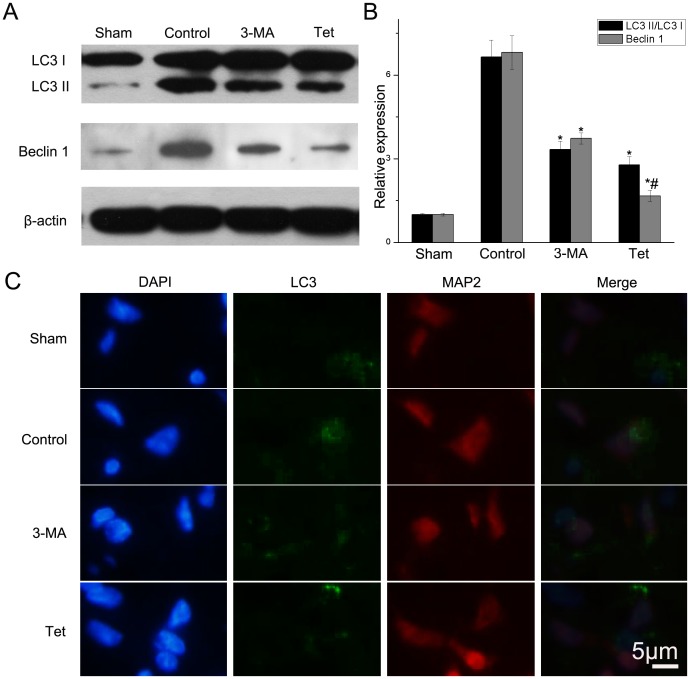
Tetracycline inhibiting the protein levels of LC3 and Beclin 1. (A) LC3 and Beclin 1 in brain tissues detected by western blotting using β-actin as loading control. (B) Relative density of LC3 II/LC3 I and Beclin 1. (C) LC3 detected by immunofluorescence. Scale bar = 5 µm. Bars represent mean±SD (n = 6); *P<0.05 vs. control group, #*P*<0.05 vs. 3-MA group.

To evaluate the activity of autophagy, the LC3 II and Beclin 1 levels were detected with western blotting analysis. Quantitative analysis revealed that ischemic stroke increased the ratio of LC3 II to LC3 I compared with sham group (6.65±0.60 vs. 1.00±0.03, *P*<0.05, Fig.1) while treatment of 3-methyladenine (3-MA, Sigma-Aldrich, USA) reversed the trend (3.33±0.31 vs. 6.65±0.60, *P*<0.05, Fig.1). The ration of LC3 II to LC3 I in the Tet group in which rats were given tetracycline daily one week prior to experimental stroke was also down regulated compared with the control group (2.78±0.31 vs. 6.65±0.60, *P*<0.05, Fig.1). Consistent with LC3, Beclin 1 was up regulated after ischemia (*P*<0.05). When compared to the control group, Beclin 1 in the 3-MA group and Tet group tended to decrease (*P*<0.05).

### 2.2 Tetracycline Inhibited Activation of NF-κB Pathway

**Figure 2:**
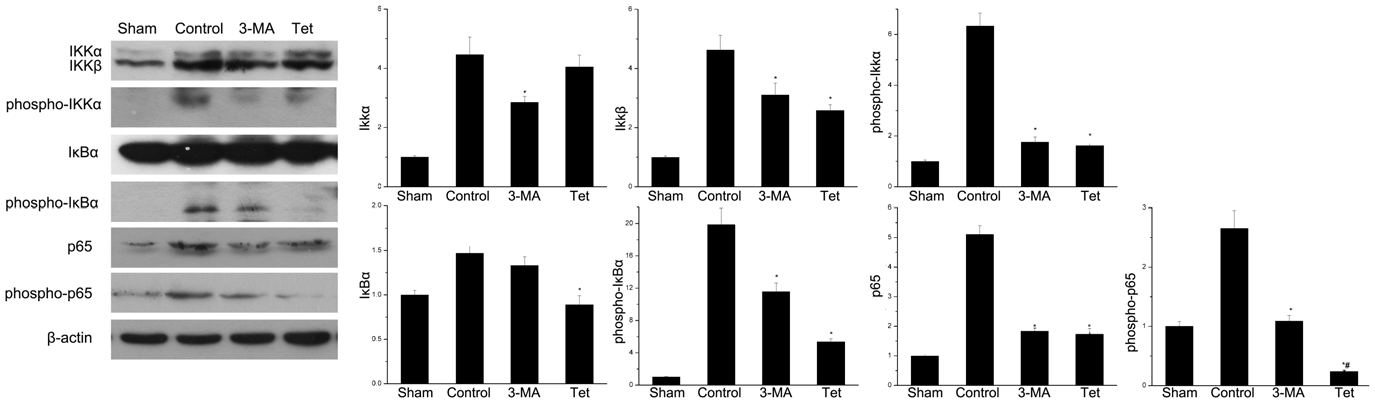
Suppression of NF-κB pathway by tetracycline in the ischemic brain. (A) The protein expressions of IKKα/β, phosphor-IKK, IκBα, phosphor-IκBα, p65 and phosphor-p65 were detected by western blotting using β-actin as loading control. Graphic representations of the ratios (B) IKKα/β-actin (C) IKKβ/β-actin (D) phosphor-IKK/β-actin (E) IκBα/β-actin (F) phosphor-IκBα/β-actin (G) p65/β-actin (H)phosphor-p65/β-actin. Bars represent mean±SD (n = 6); *P<0.05 vs. control group, #*P*<0.05 vs. 3-MA group.

To investigate whether tetracycline inhibited NF-κB pathway, the key protein levels of the NF-κB pathway were measured by western blotting. Tetracycline significantly decreased the total and phosphor-p65 (*P*<0.05, Fig.2A, G, H).The total p65 and its phosphorylation were also decreased in the 3-MA group. Compared with the control group, tetracycline significantly inhibited the expression of total IκB-α (*P*<0.05, Fig.2A, E) and the phosphor-IκB-α was decreased by 73.0% (n = 6, *P*<0.05, Fig.2A, F). Interestingly, there was no significant difference of the total IκB-α between in the 3-MA group and control group (1.46±0.08 vs. 1.32±0.10, *P*>0.05, Fig.2A, E) though 3-MA significantly decreased the phosphorylation of IκB-α (11.55±1.12 vs. 19.85±2.01, *P*<0.05, Fig.2A, F). As for IKKα/β, 3-MA inhibited the expression of IKK-α (*P*<0.05, Fig.2A, B), while tetracycline significantly decreased the level of IKKβ (*P*<0.05, Fig.2A, C). Both 3-MA and tetracycline inhibited the phosphorylation of IKK (*P*<0.05, Fig.2A, D).

### 2.3 Tetracycline Decreased DNA-binding Activity of NF-κB

**Figure 3:**
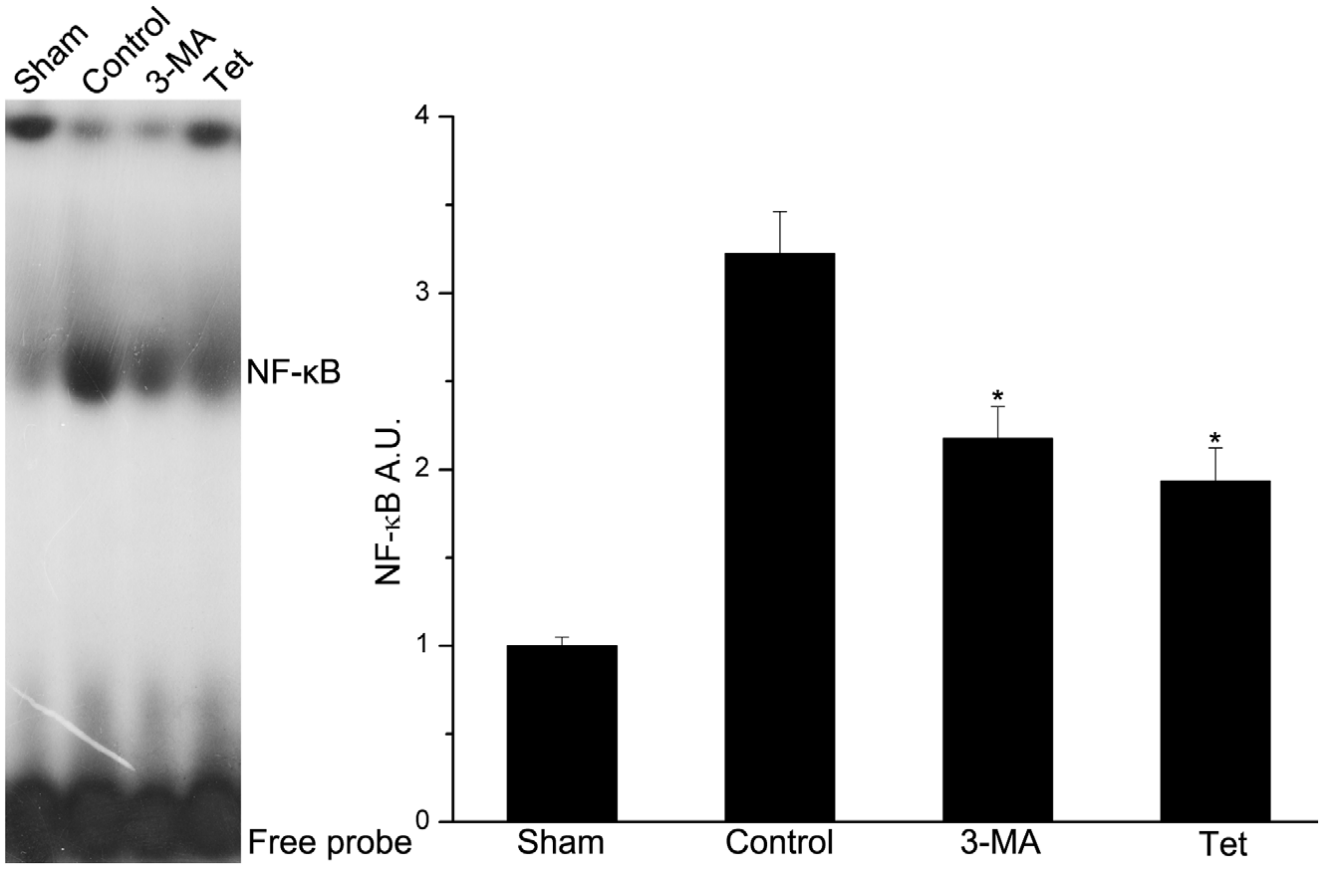
Tetracycline inhibiting the DNA binding activity of NF-κB. The binding activity of of NF-κB was assessed by Electrophoretic mobility shift assay (EMSA). (A) EMSA autoradiography of NF-κB DNA binding activity. (B) Levels of NF-κB DNA binding activity expressed as arbitrary densitometric units (A.U.) using Image J. Compared to the control group, NF-κB binding activity was significantly decreased in the tetracycline group. Bars represent mean±SD; *P<0.05 versus control group.

The DNA-binding activity of NF-κB/p65 was measured by emzyme-liked immunosorbent assay (EMSA) and expressed as arbitrary densitometric units (A.U.). In the brain tissues, ischemia increased the DNA-binding activity of NF-κB compared with the normal condition (3.22±0.24 vs. 1.00±0.05, *P*<0.05, Fig.3). And the DNA-binding activity induced by ischemia was inhibited by 3-MA inhibited (2.18±0.18 vs. 3.22±0.24, *P*<0.05, Fig.3) and tetracycline (*P*<0.05, Fig.3).

### 2.4 Tetracycline Inhibited the Expressions of TNF-α and IL 6 on Protein and mRNA Levels

**Figure 4:**
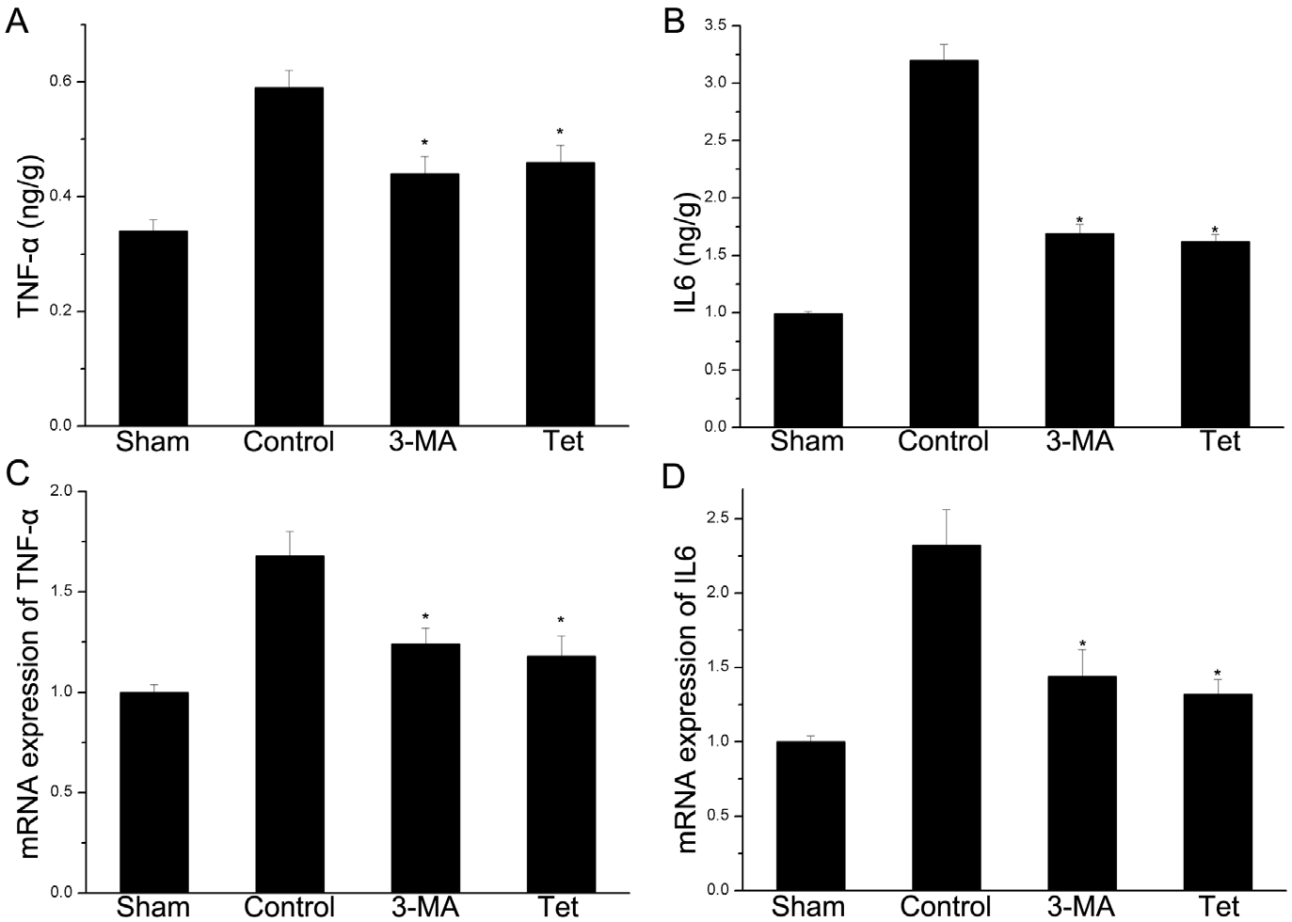
Tetracycline down-regulating the expressions of mRNA and protein of cytokines. The mRNA and protein were detected by ELISA and real time PCR respectively. (A) Both tetracycline and 3-MA significantly decreased the levels of TNF-α. (B) IL6 was significantly decreased by tetracycline and 3-MA. (C) Tetracycline down-regulated mRNA expressions of TNF-α. (B) The mRNA level of IL6 in the brain was inhibited by tetracycline and 3-MA. Bars represent mean±SD (n = 6); *P<0.05 vs. control group.

On protein level, tetracycline significantly decreased TNF-α (0.46±0.03 vs. 0.59±0.03 ng/g, *P*<0.05, Fig.4A) and IL6 (1.62±0.06 vs. 3.20±0.14 ng/g, *P*<0.05, Fig.4B) compared with the control group. The level of TNF-α and IL6 were decreased by 26.4% and 41.2% respectively in the 3-MA group when compared to control group. In consistent with the protein level, the mRNA expressions of TNF-α (1.18±0.10 vs. 1.68±0.12, *P*<0.05, Fig.4C) and IL6 (1.32±0.10 vs. 2.32±0.24, *P*<0.05, Fig.4D) were significantly decreased by tetracycline compared with the control group. The similar results were observed in the 3-MA group (Fig.4C, D). Thus, pretreatment and 3-MA decreased the infarct volume 24 h after reperfusion compared to control group (*P*<0.05, Fig.S1).

### 2.5 Tetracycline Decreased the Number of Activated Microglia in Brain

**Figure 5:**
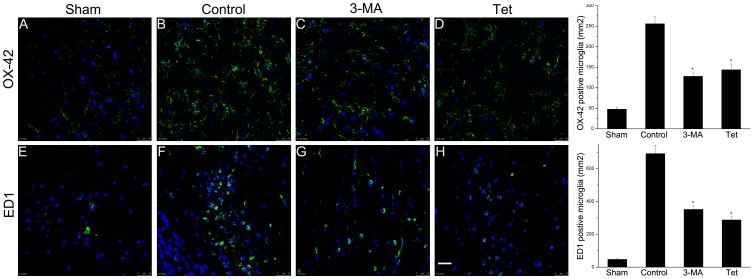
Suppressed activation of microglia by tetracycline. Activated microglia marked by OX-42 (A–D) and ED1 (E–H) were detected by immunofluorescence. Tetracycline decreased the number of activated microglia (OX-42 positive) in the brain (*P*<0.05). The number of ED1 positive microglia was also decreased (288±20 vs. 692±50/mm^2^). Scale bars: 25 µm. *P<0.05 versus control group.

Activated and phagocytotic microglia were detected by immunofluorescence using OX-42 and ED1 respectively. The numbers of OX-42 positive (256±18 vs. 48±5/mm^2^, *P*<0.05, Fig.5) and ED1 positive (692±50 vs. 48±5/mm^2^, *P*<0.05, Fig.5) microglia in the control group were increased when compared to sham group. Tetracycline significantly decreased the number of OX-42 (144±14 vs. 256±18/mm^2^, *P*<0.05, Fig.5) and ED1 positive microglia (288±20 vs. 692±50/mm^2^, *P*<0.05, Fig.5) compared with control group. Treatment of 3-MA also decreased the number of OX-42 and ED1 positive microglia (*P*<0.05, Fig. 5).

## Discussion

In this study, our results showed that pretreatment of tetracycline suppressed the activation of NF-κB pathway induced by cerebral ischemia via inhibiting the autophagy and then decreased the expressions of pro-inflammatory cytokines and activation of microglia.

Cerebral ischemia induces local inflammation and exacerbate the ischemic injury via releasing pro-inflammatory cytokines, producing free radicals and motivation of microglia [17]. TNF-α and IL6 are important pro-inflammatory cytokines. The concentration of IL 6 was in consistent with stroke severity [18], [19] and exogenous administration of TNF-α exacerbated ischemic brain injury [3]. The activated microglia caused neural damage by secreting pro-inflammatory cytokines, chemokines and adhesion molecules [4], [5]. Thus, inhibition of inflammation could provide neuroprotection. For example, inhibition of TNF-α could reduce ischemic damage [3]. Our data showed that pretreatment of tetracycline could suppress local inflammation induced by ischemia by reducing the expressions of pro-inflammatory cytokines and the activation of microglia; thus, tetracycline decreased the infarct volume (Fig. S1). These results were consistent with previous reports. In clinical trials, doxycycline treatment resulted in a profound suppression of IL6 [20] and TNF-α [21]. Jantzie et al found that administration of doxycycline could attenuate the ischemic injury via inhibiting the activation of microglia [22]. The similar results were also archived by another group [23]. However, microglia might provide neuroprotection via eliminating extracellular excess excitotoxins and engulfing the infiltrating neutrophils [24]. This disparity might be explained by the difference of time and origin [1]. The mechanisms that microglia were activated by enhanced autophagy were not completely clear. The possible reason was that autophagy was that autophagy induced by ischemia may trigger cell death by excessive self-digestion [25] and the dead cell which in turn activated microglia [26].

**Figure 6:**
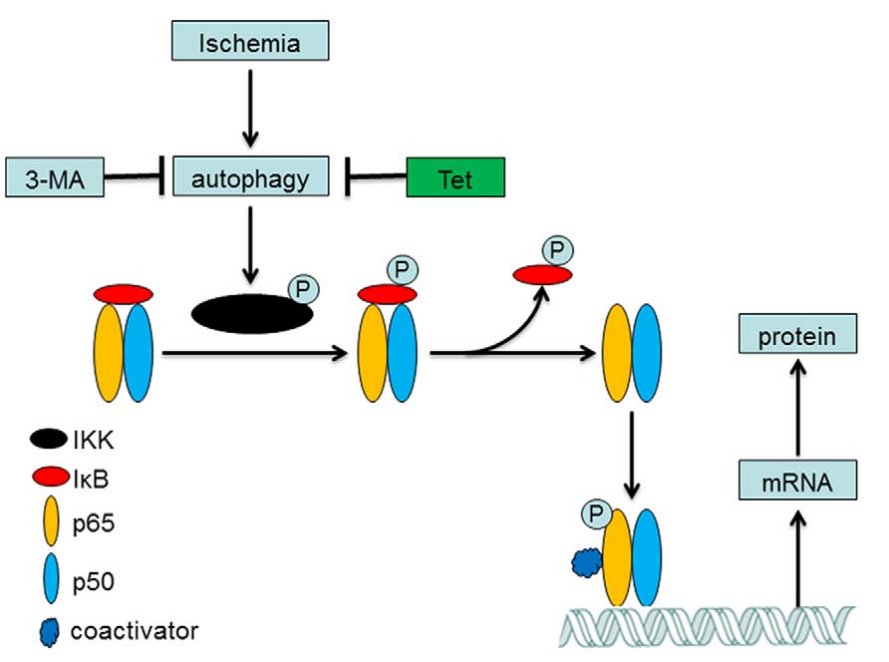
Tetracycline inhibiting the autophagy to suppress the activation of NF-κB pathway. Ischemia could induce the autophagy which in turns activated the IKK via phosphorylation. The activated IKK phosphorylated the IκBα to liberate NF-κB and allow it to translocate to the nucleus. The phosphorylated NF-κB binds to its binding sites in the target genes to initiate their transcriptions. These proteins played essential roles in inflammatory process.

The NF-κB pathway is involved in the central role of inflammatory regulation [27]. The classical pathway of NF-κB is complex; briefly, NF-κB (p65, RelB, RelC, p50 and p52) are sequestered in the cytoplasm bound to its endogenous inhibitor proteins, IκBs. Phosphorylation of IKK leads to IκB phosphorylation-dependent degradation which in turns liberates NF-κB and allows it to translocate to the nucleus, binds to specific sites and activates the target gene expression such as TNF-α and IL6 (Fig.6) [28], [29]. Through this way, NF-κB promotes neuron death in ischemic brain [30]. In the present study, we initially demonstrated that ischemia increased the expression and phosphorylation of IKK and then activated Ikβ to up-regulate the expression of total NF-κB/p65 and phosphorylated NF-κB/p65. The activated NF-κB binds to the specific sites which contained in the promoters of TNF-α and IL 6 [28], [31] and then increased their mRNA and protein expressions (Fig.4). The pretreatment of tetracycline could inhibit the mRNA and protein expressions of TNF-α and IL 6 via amending the activation of NF-κB pathway. Our results were consistent with the previous reports. Cai et al reported that administration of minocycline decreased the levels of NF-κB and TNF-α in brain tissues [32]. Oxytetracycline inhibited the NF-κB activation via attenuating the phosphorylation and degradation of IκBα [33]. However, some researchers reported that doxycycline treatment gave no statistically significant change in NF-κB activation in lung tissues [34] and others said that doxycycline up-regulated the expression of IL6 via NF-κB pathway in mouse thymic epithelial cells [35].

Another interesting finding was that 3-MA could also inhibit the activation of NF-κB pathway and decrease its downstream gene expression. As a classical specific inhibitor of autophagy [36], 3-MA, one member of phosphoinositide 3-kinase (PI3K) inhibitors, suppresses autophagy at the isolation membrane or phagophore step [37]. This result indicated that inhibition of autophagy may down regulate the activation of the NF-κB pathway. Other studies confirmed our observation. Pharmacological or genetic blockade of autophagosome formation or the inhibition of lysosomal proteases decreased degradation of IκBα and lowered NF-κB target gene expression [38]. The possible mechanism was that IKK-β activity was regulated through autophagic degradation and inhibition of phosphorylation [39]. The inflammatory process such as production of cytokines (TNF-α and IL1β) was also modulated via autophagy [40].

An interesting phenomenon was that the levels of phosphor-p65 were less in Tet group than that in 3-MA group while the NF-κB binding activity was similar in these two groups. The possible reasons were that the NF-κB binding activity were determined by many issues as we showed in Fig.6 and phosphor-p65 was one of them; thus, the similar binding activity may be observed in the different phosphor-p65 levels groups.

Beclin 1, a Bcl-2 interacting protein, is a critical component in the PI3K and plays a crucial role in the regulation of the autophagosomes formation [41]. LC3 is normally located in the cytoplasm and concentrated in autophagosomes during autophagy switching from 18 kd (LC3 I) to 16 kd (LC3 II) which could be detected by immunoblot analysis [42]. We provided the evidences that pretreatment of tetracycline down-regulated the Beclin 1 level and suppressed the conversion of LC3. The underlying mechanism may be that tetracycline could inhibit the activation of PI3K pathway. Yao et al demonstrated that minocycline inhibited the angiogenesis via down-regulating the activation of PI3K pathway [43]. This result was further confirmed by other researches [44]. However, one group found that minocycline could induce the autophagy in the cancer cells [45]. The possible reason may be that the process depends on the type of cells, time and model of diseases.

In summary, our results suggested that pretreatment of tetracycline may inhibit the activation of autophagy in ischemic brain tissues, which in turns suppressed the inflammatory process via down-regulating the activation of NF-κB pathway.

## Materials and Methods

### 4.1 Animals

This study was carried out in strict accordance with the recommendations in the guideline published in the NIH guide for the Care and Use of Laboratory Animals (National Institutes of Health Publication No. 80-23, revised 1996). The protocol was approved by the Animal Care Committee (Institute of Science and Technology, Jiangsu Province, PR China). Adult male Sprague-Dawley rats (220–250 g) were obtained from Model Animal Research Center of Jinling Hospital (Nanjing, PR China). The rats were housed under controlled environmental conditions with ambient temperature of 25°C, relative humidity of 65%, and 12/12 h light-dark cycle. Food and water were provided *ad libitum*. All surgery was performed under sodium pentobarbital (Sigma-Aldrich, USA) anesthesia, and all efforts were made to minimize the number of animals and their sufferings.

### 4.2 Experimental Protocols

Rats were randomized into four groups each of which includes six animals, sham group, control group, 3-MA group and tetracycline (Tet) group. The treatments of these groups were demonstrated in Table 1. The phosphate buffered saline (PBS, pH 7.4, 4 µl) and 3-MA (50 nM diluted in 4 µl PBS) were given by stereotaxic injection. The rats in the Tet group were fed a diet containing tetracycline at 6 g/kg (Sigma-Aldrich) while the rats in other groups were give a standard diet. After 7 days of feeding, they were undergoing the surgery.

**Table 1:** Treatments of different groups.

Group	Treatments			
	MCAO	3-MA	PBS	Tetracycline
Sham	−	−	+	−
Control	+	−	+	−
3-MA	+	+	−	−
Tet	+	−	+	+

MCAO, middle cerebral artery occlusion; 3-MA, 3-methyladenine; PBS, phosphate buffered saline.

### 4.3 Drug Delivery and Focal Cerebral Ischemia and Reperfusion

The procedure of drug delivery was done as described before [46]. Anesthetized rats were placed in a stereotaxic frame. Stereotaxic injections were made by Hamilton syringe using the following coordinates: 3 mm rostral to bregma, 2 mm lateral to midline and 2 mm ventral to the skull surface. At the end of injection, the needle was left in place for 5 min before being slowly withdrawn. After drug delivery, focal cerebral ischemia and reperfusion was done immediately as described previously [47]. Briefly, a 4-0 silicone-coated nylon filament was advanced from the external carotid artery into the lumen of internal carotid artery until the rounded tip reached the entrance to the middle cerebral artery. A laser Doppler flow (LDF; Perimed PF5000, Stockholm, Sweden) meter was used to confirm the decrease of the middle cerebral artery blood flow immediately after the occlusion to about 20% of the basic cerebral blood flow. Following MCAO, the rats were returned to their cages. Two hours later, the animals were anesthetized again and the filament withdrawn. Rectal temperature was maintained at 37°C using a heat pad during the surgery. The animals in the sham group underwent the process of surgery without the insertion of filament.

### 4.4 Tissue Processing

Rats were sacrificed under deep anesthesia 24 h after reperfusion. For real-time PCR, ELISA, EMSA and western blotting, rats were perfused transcardially with 0.1 M PBS (pH 7.4) only, and brains were removed rapidly and stored in the liquid nitrogen until use; for immunohistofluorescence, rats were perfused transcardially with 0.1 M PBS (pH 7.4) followed by a fixative solution containing 4% paraformaldehyde in 0.1 M PBS (pH 7.4). The brains were removed and fixed in the same fixative for additional 6–12 h at 4°C. Prior to cytosectioning, tissues were cryoprotected using 20% sucrose in phosphate-buffered for 24 h followed by 30% sucrose in phosphate-buffered for another 48 h.

### 4.5 Immunohistofluorescence

All sections were processed simultaneously to ensure identical staining conditions. After rinsing in PBS (pH 7.4), the sections were incubated at 37°C for 2 h with the primary antibody in PBS used at the following dilutions: anti-OX-42 (1/200), anti-ED-1 (1/200) (AbD Serotec, UK), anti-LC3 B (1/200), anti-MAP2 (1/200) (Cell-signaling Tech.). After 4 washes with PBS, antibody visualization was achieved by the incubation at 37°C for 30 min with Alexa Fluor 488-conjugated donkey anti-mouse (1/200, Invitrogen, USA) or Alexa Dylight™–594 conjugated Goat anti-rabbit (1/200, Jackson ImmunoResearch, USA). Negative controls were prepared by omitting the primary antibodies. The sections were then cover slipped with a fluorescent mounting medium. Sections were stored at 4°C until viewing. The sections were viewed under a Leica SP5 confocal microscope (Leica, France).

### 4.6 Enzyme-linked Immunosorbent Assay (ELISA)

Brain homogenates were obtained from the ischemic hemisphere and centrifuged at 14000 g for 5 min to remove cellular debris. The supernatant was stored at −80°C until use. The concentrations of IL6 and TNF-α were measured using specific ELISA kits according to the manufacturers’ instructions (R&D system, USA).

### 4.7 Real-time Quantitative PCR

Total RNA was isolated from frozen ischemic hemisphere using the TRIzol reagent (Roche, USA) following the manufacturer’s recommendations, subjected to DNase (Promega, USA) treatment. Reverse transcript-reaction (RT) was carried out using the Firststrand cDNA synthesis kit (Fermentas, Czech) following the manufacturer’s recommendations. Obtained cDNA were amplified using the following primers: for TNF-α, 5′- GCATGATCCGAGATGTGGAA-3′ and 5′-AGACACCGCCTGGAGTTCTG-3′; for IL6, 5′- CCCACCAGGAACGAAAGTCA-3′ and 5′- GGCAACTGGCTGGAAGTCTCT-3′; for β-actin, 5′-GACAGGATGCAGAAGGAGATTACT-3′ and 5′-TGATCCACATCTGCTGGAAGGT-3′. The amplification and data acquisition were run on a real time PCR system (Agilent, USA) using SYBR green PCR Master Mix (Roche). The conditions were pre-denaturation at 95°C for 10 min, followed by 40 cycles at 95°C for 1 min and 55°C for 1 min followed by a dissociation stage at 95°C for 1 min, 55°C for 30 sec and 95°C for 30 sec. All samples were analyzed in triplicates in three independent experiments. Reactions without cDNA were used as no template control and no RT controls were also set up to rule out genomic DNA contamination. Relative quantification of mRNA expression was determined using the comparative *C*_t_ method.

### 4.8 Electrophoretic Mobility Shift Assay (EMSA)

Nuclear extracts were prepared by hypotonic lysis followed by high salt extraction. EMSA was performed using a kit (Gel Shift Assay System, Promega, USA) to assay NF-κB DNA-binding activity. The NF-κΒ oligonucleotide probe, 5′-AGTTGAGGGGAC TTTCCCAGGC-3′, was end-labeled with [γ-^32^P]. Protein-DNA binding assays were performed with 80 µg of nuclear protein. The binding medium contained 4% glycerol, 1% NP40, 1 mM MgCl_2_, 50 mM NaCl, 0.5 mM EDTA, 0.5 mM DTT, and 10 mM Tris/HCl (pH 7.5). Sample were incubated at room temperature for 15 min, and the nuclear protein with ^32^P-labeled oligonucleotide complex was separated from free ^32^P-labeled oligonucleotide by electrophoresis through a 4% native polyacrylamide gel in 0.5× TBE. After separation was achieved, the gel was dried (80°C, 30 min) and exposed to X-ray film (Fuji Hyperfilm) at −80°C with an intensifying screed.

### 4.9 Western Blotting

Cortical tissues were dissected and homogenized in RIPA buffer (Cell-Signaling Tech.). Protein concentrations were determined using a BCA kit (Pierce, USA). Equal amounts of protein were separated by 12% (for LC3) or 10% (for Beclin 1, IKappa B, phosphate IKappa B) or 8% (for NF-κB, phosphate NF-κB, IKKα, IKKβ and phosphate IKKα/β) SDS-PAGE (Bio-Rad), and then transferred to 0.22 or 0.45 µm PVDF membranes (Millipore, USA) using a Trans-Blot semidry system (Bio-Rad). The membranes were blocked in 5% BSA in Tris-buffered saline with Tween 20 buffer (TBST) for 2 h, and then incubated overnight at 4°C with the following primary antibodies: anti-LC3 (1∶1000, Cell-signaling Tech.), anti-beclin 1 (1∶1000, Abcam, UK), IKappa B, phosphate IKappa B, NF-κB, phosphate NF-κB, IKKα, IKKβ and phosphate IKKα/β (1∶1000, Cell-signaling Tech.) and anti-β-actin (1∶1000, Cell-signaling Tech.), which served as a loading control. Then the membranes were washed 3 times with TBST and incubated with horseradish peroxidaseconjugated secondary antibody (goat anti-rabbit IgG, 1∶2000 or goat anti-mouse IgG, 1∶1000, Cell-signaling Tech.) for 1 h under room temperature. Blots were developed using a chemiluminescence kit (Millipore) and exposed to X-ray film. The bands on the film were scanned and analyzed with Quality One (Bio-Rad).

### 4.10 Cell Counting

Five sections, 2 mm interval from bregma, were used for immunohistofluorescene counting in each rat. Counting was performed on six randomly selected non-overlapping per section. Design-based stereology and systematic random sampling were employed to ensure accurate and non-redundant cell counting. Every section under analysis was a minimum distance of 150 µm from the next. The number of cells was quantified using Image-Pro software 6.0. Cell courts were performed without knowledge of experimental treatments.

### 4.11 Statistical Analysis

Statistical data were shown as mean±SD. The significance of differences was determined using t test and analysis of variance (ANOVA) followed by post hoc t test using SPSS13.0 software. Statistical significance was defined as *P*<0.05.

## Supporting Information

Figure S1 **Infarct volume decreased by treatment of tetracycline and 3-MA.** The infarct volume was determined by TTC staining 24 h after reperfusion. The infarct area of each brain was measured in a blinded manner using Image J (NIH, USA). The infarct volume was then calculated by Swanson’s method. The infarct volume was decreased in Tet and 3-MA group when compared to control group. *P<0.05 versus control group.
(TIFF)
