# Supplementary figures and images for: Tetracycline Inhibits Local Inflammation Induced by Cerebral Ischemia via Modulating Autophagy
Source: PLoS One. 2012 Nov 7;7(11):e48672. doi: 10.1371/journal.pone.0048672 (PMC3492486; doi:10.1371/journal.pone.0048672)

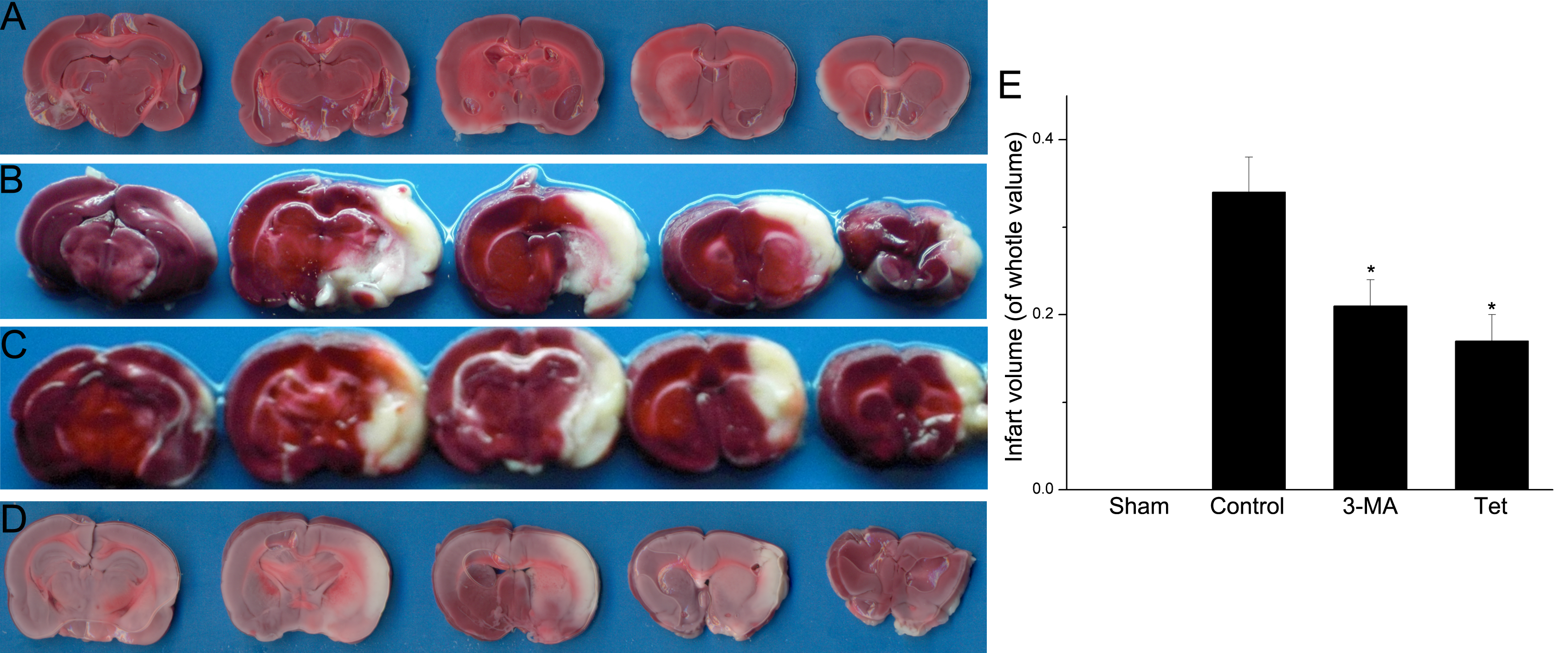

Supplement: Figure S1 — Infarct volume decreased by treatment of tetracycline and 3-MA. The infarct volume was determined by TTC staining 24 h after reperfusion. The infarct area of each brain was measured in a blinded manner using Image J (NIH, USA). The infarct volume was then calculated by Swanson’s method. The infarct volume was decreased in Tet and 3-MA group when compared to control group. *P<0.05 versus control group. (TIFF) [file pone.0048672.s001.tiff]
